# Miniaturized Broadband Bi-Yagi Antenna Array for Ambient RF Energy Harvesting

**DOI:** 10.3390/mi14122181

**Published:** 2023-11-30

**Authors:** Zaed S. A. Abdulwali, Ali H. Alqahtani, Yosef T. Aladadi, Majeed A. S. Alkanhal, Yahya M. Al-Moliki, Khaled Aljaloud, Mohammed Thamer Alresheedi

**Affiliations:** 1 Department of Electrical Engineering, King Saud University, Riyadh 11421, Saudi Arabia; zabdulwali@ksu.edu.sa (Z.S.A.A.); majeed@ksu.edu.sa (M.A.S.A.); yalmoliki@ksu.edu.sa (Y.M.A.-M.); malresheedi@ksu.edu.sa (M.T.A.); 2 Department of Applied Electrical Engineering, Al-Muzahimya Campus, College of Applied Engineering, King Saud University, Riyadh 11421, Saudi Arabia; kaljaloud@ksu.edu.sa

**Keywords:** energy harvesting, broadband antenna, miniaturized antenna, Bi-Yagi antenna array

## Abstract

This paper presents a miniaturized broadband Bi-Yagi antenna array that covers a bandwidth from 1.79 GHz to 2.56 GHz. The proposed antenna achieves a tradeoff between maximizing bandwidth, effective area, and gain while minimizing physical dimensions. The antenna design considers the coupling between the radiator and director elements, resulting in increased bandwidth as the resonating modes shift apart. Additionally, the proposed design optimizes element spacing and dimensions to achieve high gain, wide bandwidth, efficient radiation, and a minimum aperture size. The proposed antenna, with physical dimensions of 138.6 mm × 47.7 mm × 1.57 mm, demonstrates gains ranging from 6.2 dBi to 9.34 dBi across the frequency range, with a total efficiency between 88% and 98%. The proposed design is experimentally validated by measuring the reflection coefficients, input impedance, gain, and normalized radiation pattern. These features make the antenna well suited for capturing and harvesting electromagnetic waves in mobile wireless and Wi-Fi applications.

## 1. Introduction

The field of electromagnetic energy harvesting has gained significant recognition as a promising technology, aiming to offer sustainable and dependable power sources across a wide range of IoT applications [1,2,3,4,5]. The exceptional versatility of RF energy harvesting has facilitated its successful integration into wireless sensor networks, Internet of Things (IoT) devices [3], and wearable electronics [4], thereby significantly extending the lifespan and enhancing the functionality of these devices. However, many challenges should be considered to improve the performance of the rectenna since the extremely low ambient EM waves of different RF sources operate in various frequency ranges. Some of these challenges are related to the antenna used in the RF energy harvesting system. For instance, the gain and bandwidth should be designed to capture energy effectively across a wide frequency band to maximize RF energy harvesting. Moreover, the antenna size should be as small as possible to fit specific IoT applications. Furthermore, the complexity of the antenna structure should be considered for economic purposes. Review studies and comparative analyses of antenna designs for RF energy harvesting are presented in [5,6,7]. The gain, bandwidth, size, and complexity are considered as the main features for comparisons. Unfortunately, improving one of these features results in the deterioration of others [8]. Therefore, some researchers focus on minimizing the antenna structure, as in [9,10,11]. The main drawback of such a structure is the low gain and narrow bandwidth. However, these antennas can be used for applications of wireless power transfer where an intended source is used for transmitting RF power from a near distance, or for applications that have critical size and weight limitations. On the other hand, some researchers have tried to use wide bandwidth antennas to harvest energy from different ambient sources. Multiple bands or wide bands antennas have been developed, as in [12,13,14,15,16]. For instance, in [16], an array of 64- broadband elements is used for ambient energy harvesting with DC-combining techniques. However, these efforts are usually achieved by sacrificing antenna element gain.

Considerable efforts have been made to improve antenna gain since the ambient incident signal is too low to drive the rectifier circuitry [17,18,19]. Similar effort goes into maximizing antenna gain using metasurfaces. For instance, metasurfaces are employed as superstrates to enhance antenna gain [20,21]. In [22,23,24,25,26,27], periodic structures with small resonators compared to the operating frequency are connected to load resistances or rectifiers. However, the large number of ports on the metasurfaces can lead to high costs and power losses. To overcome this challenge, only the center cell/supercell is utilized for power harvesting while treating the other cells as parasitic [28,29]. Furthermore, investigations have been conducted on the use of one-port or two-port metasurfaces in electromagnetic energy harvesting, studying their performance in both transmitting and receiving scenarios [23,30,31,32]. However, the large size and limited bandwidth of such structures make this category less effective for some IoT applications. Similarly, in [33], a two-port grid-array antenna is used to provide a rectenna with different beam angles and beamwidths to overcome direction problem.

Furthermore, extensive research efforts have been dedicated to the pursuit of integrating multiple features within a single structure. This approach frequently involves incorporating two or three elements or utilizing multiple ports, with the intention of improving not only gain, directionality, and bandwidth, but also the overall performance of the system [34,35,36]. In [17], a Yagi array is presented which has great gain, and the bandwidth covers the extended GSM 1800 and UMTS 2100 bands. However, the relative bandwidth is only 18%, which does not include the ISM Wi-Fi band, and the size is not perfectly minimized. In [18], a compact collinear quasi-Yagi array is presented. The array has good gain and size, but the relative bandwidth is only 13% and does not include the extended GSM band. For RF energy harvesting, it is good to have an antenna structure that has a wide bandwidth to include most possible ambient sources. Therefore, a tradeoff between bandwidth, gain, and size needs to be carefully considered in RF energy harvesting design.

This paper introduces a miniaturized broadband Bi-Yagi antenna array design that effectively operates within a wide frequency range from 1.79 GHz to 2.56 GHz to be used for electromagnetic energy harvesting. The proposed design is experimentally validated by measuring the reflection coefficients, input impedance, and normalized radiation pattern. The antenna design achieves a well-balanced tradeoff between maximizing bandwidth, effective area, and gain, and minimizing the physical dimensions. By considering the coupling effects between the radiator and director elements, the antenna’s bandwidth is significantly enhanced as the resonating modes experience distinct separation. Additionally, through a careful optimization of the element spacing and dimensions, the antenna demonstrates impressive performance characteristics, including high gain, wide bandwidth, efficient radiation, and a compact aperture size.

## 2. Antenna Design and Analysis

When evaluating electromagnetic energy harvesting antennas, several parameters are crucial for assessing their performance. The effective area, which determines the power received by the antenna, is a fundamental consideration. It is directly proportional to the power density of the incident electromagnetic wave and can be enhanced by optimizing the antenna dimensions, polarization, radiation pattern, and impedance matching. Gain plays a vital role in amplifying the received signal, enabling the antenna to capture more energy from the ambient electromagnetic field. Efficiency is equally significant, representing the ability to convert the captured energy into usable electrical power with minimal losses. Maximizing these parameters collectively leads to improved energy harvesting capabilities and overall system performance. The receiving antenna’s effective area $A_{eff}$ can be calculated using antenna transmitting parameters as in Equation (1) [37]

(1) $$A_{eff}=\frac{{}^{2}G}{4\pi }$$

where λ is the wavelength of the incident wave, and *G* is the antenna gain. Working at low frequencies and increasing the antenna gain will increase the effective area of the receiving antenna, assuming perfect matching with load and polarization. On the other hand, integrating this power at a different frequency will increase the amount of harvested power. Thus, the another important parameter is the bandwidth. Both are related to the structure size and radiation technique. A microstrip antenna is commonly used in energy harvesting systems due to its profile advantages. The substrate of the microstrip antenna should be chosen wisely to improve radiation and matching characteristics [37,38,39]. In particular, a low-loss tangent (*δ*) substrate enhances efficiency and gain whereas a higher loss tangent broadens the bandwidth [38]. However, bandwidth can be increased using different ways while it is difficult to compensate loss, especially in RF energy harvesting. On the other hand, it is better for the dielectric-constant (*ε_r_*) to be low to improve bandwidth. However, a higher *ε_r_* substrate will be good for the minimization of the resonance length of the antenna. Another important parameter to consider in antenna design is the substrate height. Increasing the substrate height enhances efficiency (ignoring surface wave effects) and antenna bandwidth. However, increasing the substrate height can lead to the emergence of undesired surface waves, particularly for substrates with dielectric constants greater than unity. These surface waves propagate within the substrate and experience scattering at bends and surface irregularities, such as dielectric and ground truncations. As a result, this scattering process negatively impacts the antenna’s pattern and polarization properties, leading to degradation [38,39]. Therefore, minimizing tangential loss is crucial for increasing radiation efficiency. The substrate should be made as thick as possible, considering the emergence of surface waves and spurious radiation that may impact polarization purity. Additionally, a small dielectric constant improves antenna bandwidth. Therefore, a substrate with a thickness of 1.57 mm, a dielectric constant *ε_r_* of 2.2, and a loss tangent (*δ*) of 0.0009 is selected for the proposed structure. This choice minimizes surface waves, reduces power losses, enhances polarization purity (i.e., low cross-polarization), and increases bandwidth.

### 2.1. Three-Element Yagi Antenna Geometry Development

**Figure 1:**
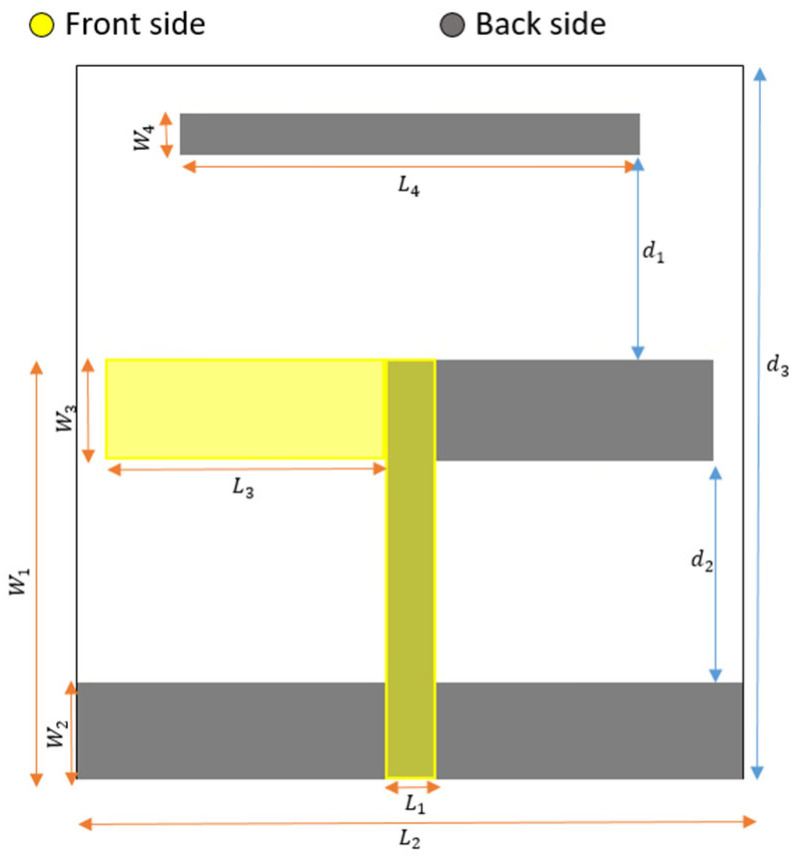
Three-element printed Yagi antenna.

The structure of the three-element printed Yagi antenna and its main important parameters are shown in Figure 1. It is etched on a 138.6 mm × 47.7 mm Roger RT/duroid 5880 dielectric of 1.57 mm thickness. One arm of the dipole radiator is printed on the front side, while the other arm is printed on the back side of the dielectric substrate. The twin-lead transmission line is used to feed this radiator, as depicted in Figure 1. Moreover, the reflector and director are also printed on the back side of the dielectric substrate.

The presence of the substrate causes a detuning effect on the dipole, reflector, and director, resulting in shorter lengths compared to their counterparts in free space. The guided wavelength used to calculate the preliminary lengths is given by Equation (2) and fed by a microstrip line of width *W* = 4.9 mm to have 50 Ω matching [37].

(2) $$\lambda_{g}=\;\frac{\lambda_{0}}{\sqrt{\varepsilon_{reff}}}\;$$

where $\lambda_{0}$ is the free space wavelength and *ε_reff_* is the effective dielectric constant given by Equation (3)

(3) $$\varepsilon_{reff}=\frac{\varepsilon_{r}+1}{2}+\frac{\varepsilon_{r}-1}{2}{\left[1+12\frac{h}{w}\right]}^{-\frac{1}{2}}$$

where $\varepsilon_{r}$ is the dielectric constant and *h* is the substrate thickness.

**Figure 2:**
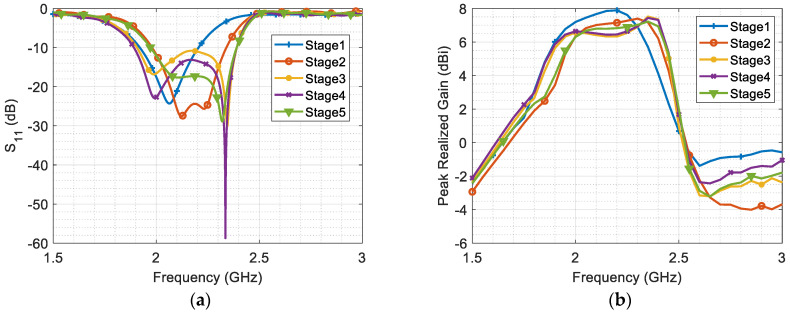
(**a**) The reflection coefficient S_11_, and (**b**) the realized gain in dBi for five stages.

The resulting effective dielectric constant is approximately 1.87, and at a frequency of 2 GHz, the guided wavelength (*λ_g_*) measures 109.6 mm. First, a two-element Yagi antenna is examined using a full-wave simulator. The dimensions of the derived radiator (dipole) and reflector are approximately set to 0.5*λ_g_*, with a width of 4.9 mm and a spacing of around 0.2*λ_g_* between them. Subsequently, a parametric sweep is conducted to investigate the width and spacing of the printed Yagi elements. Increasing the width of the radiator and reflector, along with their spacing, causes a slight leftward shift (i.e., decrease) in the resonant frequency, while also affecting the matching level and directivity. A tradeoff is chosen to develop a three-element Yagi antenna, where an additional parasitic element (i.e., director) is added to enhance antenna gain and bandwidth. The dimensions of the radiator (dipole), reflector, and director elements are 0.52*λ_g_*, 0.63*λ_g_*, and 0.43*λ_g_*, respectively, with a spacing of around 0.2*λ_g_* between them. The length of the reflector is chosen after parametric sweep to prevent backside radiation, avoiding unnecessary size increase in the design. The initial widths of the derived radiator, reflector, and director elements are set to approximately 0.093*λ_g_*, 0.082*λ_g_*, and 0.036*λ_g_*, respectively, while the width of the feeding line is 0.045*λ_g_*. To explore tradeoff improvements between antenna size, gain, and bandwidth, five stages are presented, each with distinct design parameter values chosen after parametric sweep, as illustrated in Table 1. The corresponding results of these antenna stages, including S_11_ and realized gain, are depicted in Figure 2a,b.

The S_11_ results in Figure 2a show that the resonant frequencies of the director and radiator are degenerated together for the first stage. However, in the second stage, as the width of the director and the spacing between the radiator and both the reflector and director are reduced, the two resonating modes become separated. Thus, the second stage demonstrates a smaller size and wider bandwidth due to increased coupling, which generates a new resonant mode at a higher frequency. However, the realized gain of the antenna decreases due to the reduced aperture length as shown in Figure 2b.

In the third stage, the width of the director is minimized significantly to enhance the coupling effect between the radiator and director, thereby increasing the current density of the second resonating mode (i.e., it is maximum on the director surface at the second resonance 2.34 GHz). The resulting coupling causes the two resonating modes to shift apart, thereby expanding the bandwidth.

In the fourth stage, two square notches with dimensions of approximately 3 mm × 3 mm are implemented on the upper-outer and lower-inner corners of the dipole arms. This stage highlights the substantial improvement in antenna matching achieved through these notches, while preserving gain and bandwidth, ultimately enhancing the overall efficiency. In the final stage, the spacing between the reflector and radiator elements is further reduced, contributing to size minimization. The coupling effect with the reflector influences the bandwidth, but the size is approximately 20% smaller than in Stage#4. Further improvements in this stage is explored in the Bi-Yagi array design.

### 2.2. Bi-Yagi Array Development

**Figure 3:**
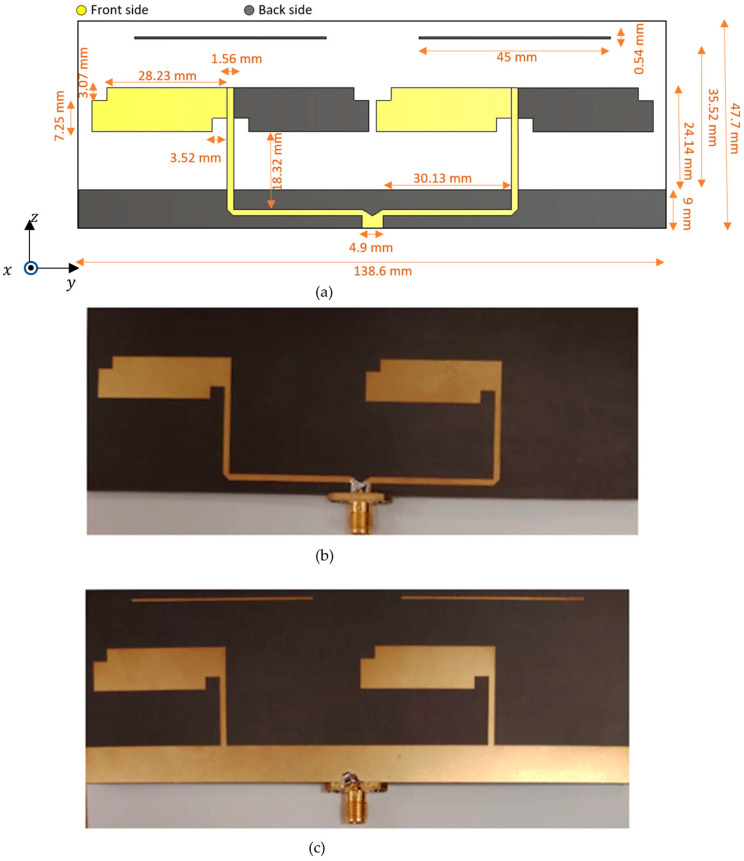
(**a**) Optimum design of Bi-Yagi antenna array, (**b**) front side of Bi-Yagi antenna array prototype, and (**c**) back side of Bi-Yagi antenna array prototype.

Using the previously developed three-element Yagi antenna, an optimal design of a Bi-Yagi antenna array is presented. Figure 3 illustrates the optimal antenna parameters and shows the front and back sides of the proposed prototype. The selection and optimization of these parameters aim to minimize the size of the antenna array while improving the bandwidth and gain. The antenna is etched on a rectangular substrate, measuring 138.6 mm × 47.7 mm, composed of Roger RT5880 material with a dielectric constant of 2.2 and an electric tangent loss of 0.0009, which enhances radiation efficiency. The antenna demonstrates excellent performance within the desired bandwidth of 1.79 GHz to 2.56 GHz, with a bandwidth of 770 MHz centered at 2.15 GHz. The decrease in distance between the two array elements increases the matching. The close spacing between the antennas enhances their mutual coupling, contributing to the constructive modification of the combined RF signal through the feeding network. This leads to an improved bandwidth. Additionally, the array achieves a compact size while maintaining satisfactory performance, which is discussed in the “Results and Discussion” section.

## 3. Results and Discussion

The antenna port is excited by a Gaussian pulse with a 50 Ω coaxial feeding port. The EM wave polarized along the *y*-axis and propagating along the +*z*-axis, as shown in Figure 3a. The design of the proposed antenna was supported by electromagnetic full-wave simulations conducted using the finite integration technique (FIT) solver in Computer Simulation Technology (CST). This full wave simulator facilitated a thorough examination of crucial parameters such as the reflection coefficient, impedance matching, gain, and radiation patterns. To validate the simulation results, a vector network analyzer (VNA) system and the Geozondas time-domain measurement setup were utilized.

**Figure 4:**
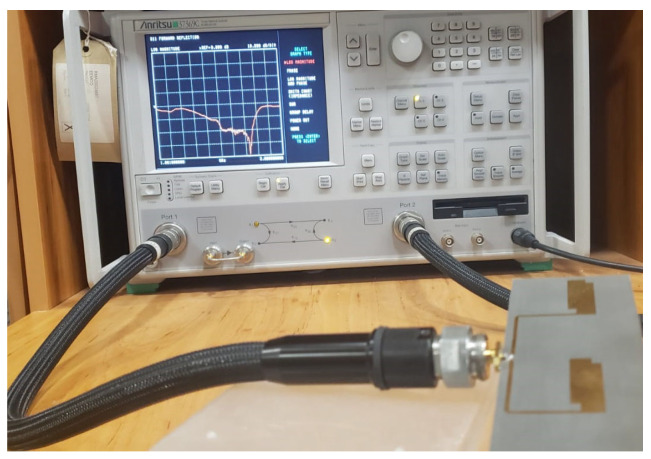
The reflection coefficient and input impedance measurement setup.

The utilization of the VNA system played a critical role in accurately measuring the antenna’s reflection coefficients and impedance matching. Figure 4 illustrates the vector network analyzer system that was utilized to measure the reflection coefficients and input impedance (Z_11_) of the fabricated antenna.

**Figure 5:**
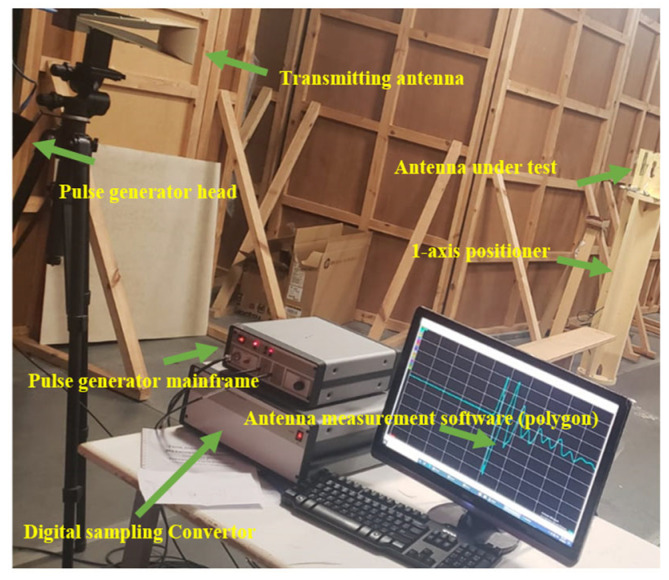
Geozondas time-domain antenna measurement setup.

Figure 5 depicts the Geozondas time-domain antenna measurement setup utilized for measuring the gain and radiation pattern of the designed antenna in the time domain. This setup incorporates several components, including a pulsed signal generator, a digital sampling converter, a transmitting antenna, a receiving antenna, and an oscilloscope. One notable advantage of this system is its ability to operate independently of a specialized environment such as a chamber room. The measurement is achieved by capturing the first line-of-sight pulsed signal and effectively eliminating other reflected pulsed signals originating from various surfaces through the adjustment of the time window. The pulsed signal generator generates short-duration pulses that are transmitted to the transmitting antenna, which subsequently radiates them into space with the main lobe directed toward the receiving antenna. The receiving antenna, which represents the antenna under test, captures the radiated signal, which is then subjected to analysis. To measure the radiation pattern, the antenna under test is positioned on a 1-axis positioner, which is controlled by the main program and permits 360-degree movement. At each selected degree, the received signal is transformed into the frequency domain using a built-in algorithm. By comparing the received signal in the frequency domain to the transmitting signal, it becomes possible to calculate the gain and radiation pattern.

**Figure 6:**
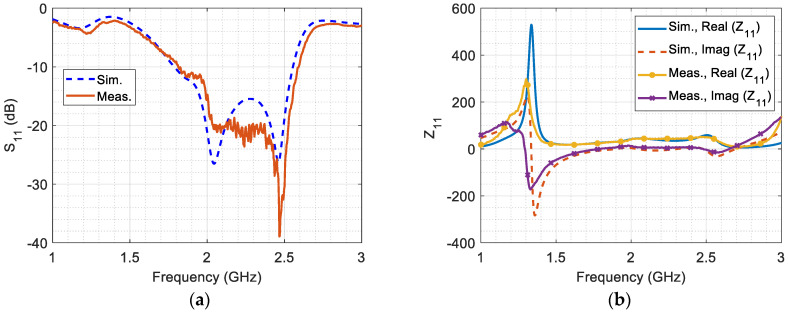
(**a**) The simulated and measured reflection coefficient, and (**b**) the simulated and measured input impedance versus frequency.

Figure 6a,b shows the impressive reflection coefficient and input impedance. It is noteworthy that the antenna exhibited excellent matching within the specified bandwidth of 770 MHz, with a central frequency of 2.175 GHz. Throughout this bandwidth, the reflection coefficients remained below −10 dB. Additionally, the real wave impedance remained consistently near 50 Ω at this frequency, while the imaginary part of the wave impedance approached zero. These results confirm the antenna’s good matching with the 50 Ω coaxial feeding port. The comparison between these measured values and the simulated results established a robust validation process, affirming the accuracy of the simulation model.

**Figure 7:**
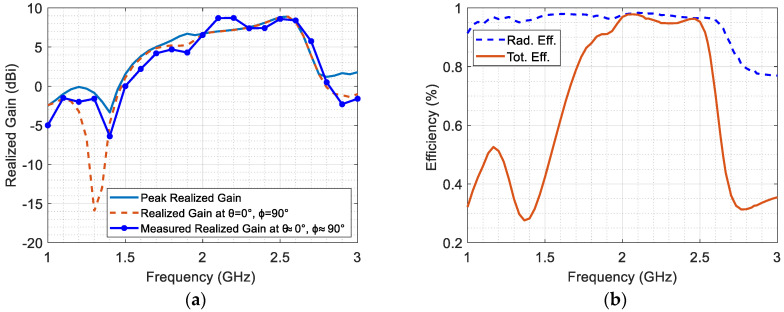
(**a**) The simulated realized gain, and (**b**) the simulated radiation and total efficiency.

Figure 7a illustrates the simulated peak realized gain and simulated realized gain at θ = 0° and ϕ = 90°, which is validated by the measured realized gain at θ ≈ 0° and ϕ ≈ 90°. The proposed antenna demonstrates a peak realized gain ranging from 6.2 dBi to 9.34 dBi across the entire frequency range. Within the selected band, the peak realized gain coincides with the gain at θ = 0° and ϕ = 90° for most frequencies. The measured realized gain at θ ≈ 0° and ϕ ≈ 90° is approximately similar to the simulated gain, with slight differences potentially arising from nonalignment.

Figure 7b illustrates the radiation efficiency and total efficiency of the antenna, highlighting its potential for electromagnetic energy harvesting. The radiation efficiency maintains a high level of around 97% across the entire bandwidth. Moreover, the total efficiency of the antenna falls within the notable range of 88% to 98% across the desired bandwidth. These remarkable performance metrics emphasize the antenna’s effectiveness in converting input power into useful radiated energy, establishing it as a promising choice for electromagnetics energy harvesting applications.

**Figure 8:**
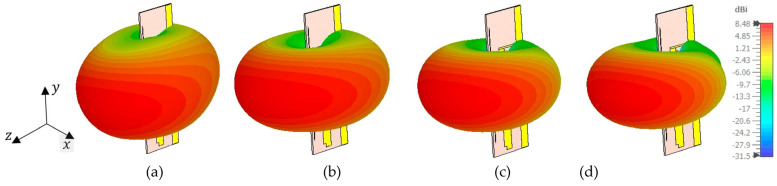
The 3D gain patterns of the proposed antenna at various frequencies: (**a**) 1.8 GHz, (**b**) 2.15 GHz, (**c**) 2.45 GHz, and (**d**) 2.55 GHz.

Figure 8 presents the 3D gain radiation pattern of the proposed Bi-Yagi antenna array. This radiation pattern illustrates the spatial distribution of radiated energy in three dimensions, specifically at the frequencies of 1.8 GHz, 2.15 GHz, 2.45 GHz, and 2.55 GHz. The red color is employed to represent the highest level of radiated energy. It is worth emphasizing that the main lobe directions consistently remain unchanged towards the z-direction across all these 3D patterns. This consistency highlights the antenna’s capability to deliver continuous and stable radiation in this particular direction.

**Figure 9:**
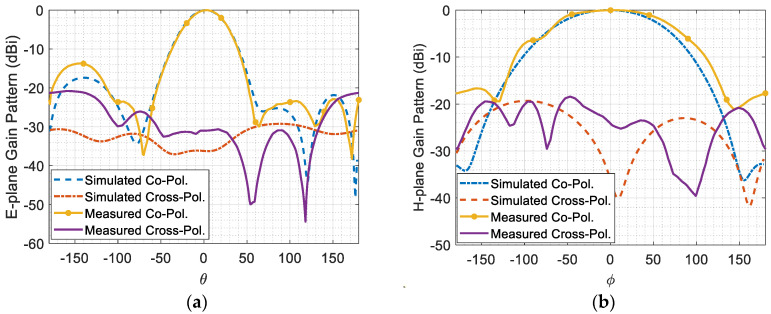
(**a**) E-plane gain radiation pattern, and (**b**) H-plane gain radiation pattern at 2.45 GHz.

Figure 9 depicts the gain radiation patterns at 2.45 GHz in both the E-plane and H-plane for both co-polarization and cross-polarization. In the context of this figure, the E-plane corresponds to the zy-plane, as depicted in Figure 7, which is perpendicular to the *x*-axis. Likewise, the H-plane corresponds to the zx-plane, which is perpendicular to the *y*-axis. It is observed that the difference between the co-polarized and cross-polarized radiation patterns in both the E-plane and H-plane is greater than around 20 dBi. This difference indicates a high level of linear polarization purity along the *y*-axis, implying that the antenna is effectively radiating in the desired polarization and minimizing radiation in unwanted orthogonal polarizations. Furthermore, it is notable that the difference between the measured and simulated data is more pronounced in the case of cross-polarization compared to co-polarization. This variation can be primarily attributed to measurement errors, for small-level signals. Cross-polarization signals are generally weaker compared to co-polarization signals, making them more susceptible to measurement inaccuracies and noise.

**Figure 10:**
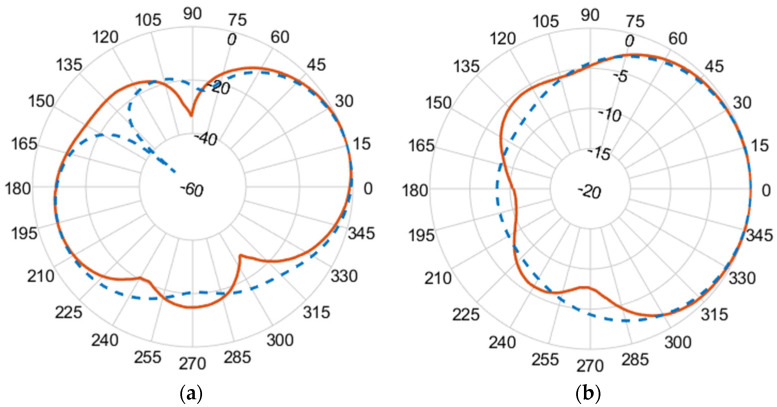
(**a**) E-plane co-polarized gain radiation pattern, and (**b**) H-plane co-polarized gain radiation pattern at 1.8 GHz.

**Figure 11:**
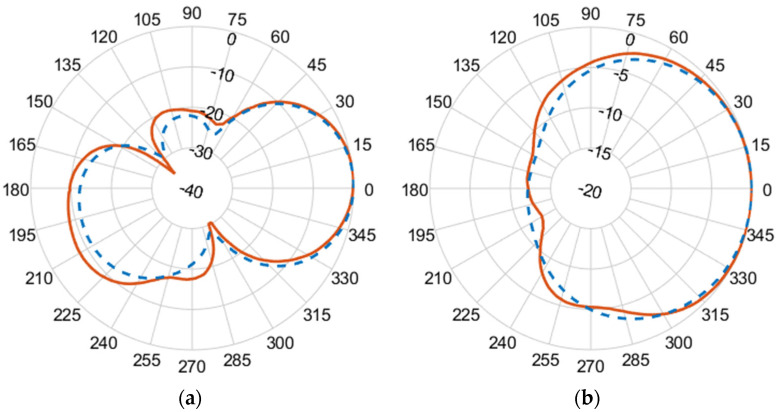
(**a**) E-plane co-polarized gain radiation pattern, and (**b**) H-plane co-polarized gain radiation pattern at 2.15 GHz.

**Figure 12:**
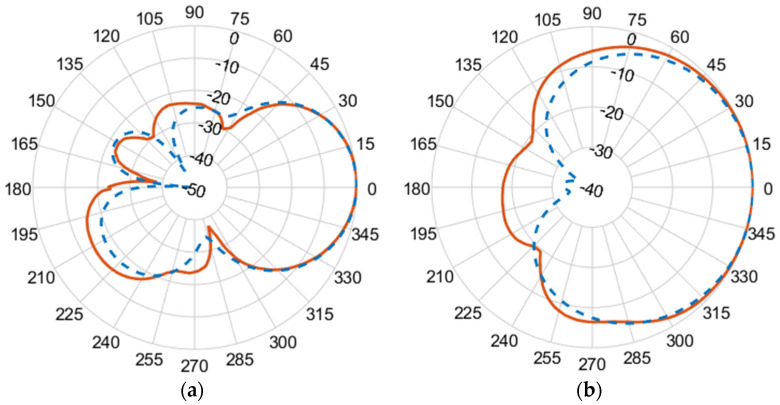
(**a**) E-plane co-polarized gain radiation pattern, and (**b**) H-plane co-polarized gain radiation pattern at 2.45 GHz.

**Figure 13:**
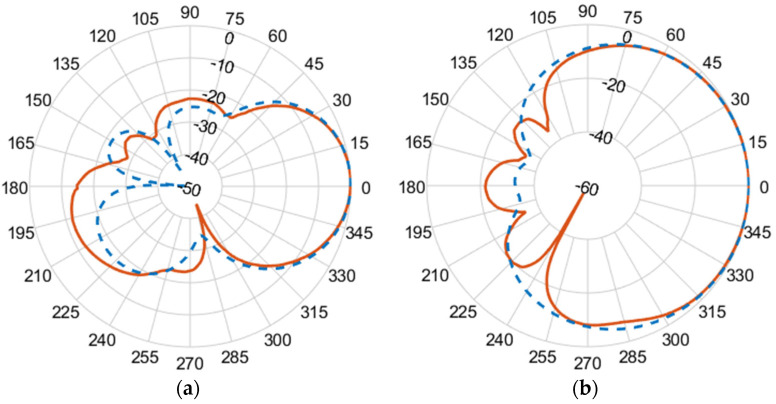
(**a**) E-plane co-polarized gain radiation pattern, and (**b**) H-plane co-polarized gain radiation pattern at 2.55 GHz.

Figure 10, Figure 11, Figure 12 and Figure 13 illustrate the simulated (dashed line) and measured (solid line) 2D normalized polar radiation patterns for both the E and H planes at specific frequencies: 1.8 GHz, 2.15 GHz, 2.45 GHz, and 2.55 GHz, respectively. These patterns provide valuable information about the antenna’s performance characteristics. It is evident that the radiation in the H plane exhibits a broad beamwidth, whereas in the E plane, it demonstrates a narrow beamwidth.

The measured co-polarized polar gain radiation patterns closely aligns with the simulated results, confirming the antenna’s consistent performance. Additionally, the antenna exhibits a favorable linear polarization pattern, enabling the efficient reception of co-polarized signals. The slight differences between the simulated and measured results in the backlobe can be attributed to the effects of the transmission line of the coaxial feeding port.

This rigorous approach, combining simulation and experimental tools, ensures a robust assessment of the antenna’s performance, accuracy, reliability, and suitability for practical applications.

Table 2 presents a performance comparison between the proposed antenna and various published works, highlighting the distinctive advantages of the proposed design. Specifically, the proposed antenna excels in achieving a balance between maximizing bandwidth, effective area, and gain, while minimizing physical dimensions, surpassing the performance of the other listed works. The design of the antenna takes into consideration element spacing and dimensions to achieve remarkable features, including high gain, wide bandwidth, and efficient radiation.

It is worth noting that the effective areas are calculated at the upper frequencies within the working bandwidth, utilizing Equation (1). The physical dimensions of the antenna aperture measure 138.6 mm × 47.7 mm × 1.57 mm. The antenna demonstrates an impressive gain range, starting from 6.2 dBi at the lower frequency of 1.79 GHz and reaching an impressive 9.34 dBi at 2.56 GHz. Notably, the effective area of the proposed antenna is specifically measured at 9.34 × 10^−3^ m^2^ at 2.56 GHz, further highlighting its exceptional capabilities and reinforcing its potential for efficient electromagnetic energy harvesting.

## 4. Conclusions

In this paper, a miniaturized broadband Bi-Yagi antenna array is proposed to cover a frequency range from 1.79 GHz to 2.56 GHz. The design of the antenna aims to strike a balance between maximizing bandwidth, effective area, and gain, and minimizing physical dimensions. Various factors are taken into consideration, including the coupling effect between the radiator and director elements, which enhances the current density of the second resonating mode. This coupling significantly enhances the current density of the second resonating mode, resulting in a notable expansion of the overall bandwidth.

Furthermore, the proposed antenna design considers the selection of element spacing and dimensions, enabling the attainment of impressive features such as high gain, wide bandwidth, efficient radiation, and a compact aperture size. The physical dimensions of the antenna aperture are 138.6 mm × 47.7 mm × 1.57 mm. The antenna exhibits a gain range starting from 6.2 dBi at the lower end of the bandwidth (1.79 GHz) and reaching 9.34 dBi at 2.56 GHz. Furthermore, the total efficiency of the antenna falls within the range of 88% to 98% across the desired bandwidth. The effective area of the proposed antenna is 9.34 × 10^−3^ m^2^ at 2.56 GHz. The proposed design is experimentally validated by measuring the reflection coefficients, input impedance, and realized gain at θ ≈ 0° and ϕ ≈ 90° and normalized radiation pattern. The antenna’s characteristics make it highly suitable for the efficient capturing and harvesting of electromagnetic waves in various applications, including mobile wireless and Wi-Fi systems.

## Figures and Tables

**Table 1:** Antenna design parameters in mm.

	$L_{1}$	$W_{1}$	$L_{2}$	$W_{2}$	$L_{3}$	$W_{3}$	$L_{4}$	$W_{4}$	$d_{1}$	$d_{2}$	$d_{3}$
**Stage#1**	4.9	43.81	70	9.8	29.3	10.25	48.2	4.08	22	23.73	71.55
**Stage#2**	4.9	33.25	70	9.8	29.3	10.25	48.2	2.7	11	13.2	57.7
**Stage#3**	4.9	41.97	70	9.8	29.3	10.25	48.2	0.4	11	21.92	57.7
**Stage#4**	4.9	41.97	70	9.8	29.3	10.25	48.2	0.4	11	21.92	57.7
**Stage#5**	4.9	33.25	70	9.8	29.3	10.25	48.2	0.51	11	13.2	47.7

**Table 2:** Comparison of antenna performance with the literature.

Ref.	Freq. (GHz)	Relative Bandwidth (S11 < −10)	Maximum Gain (dBi)	Dimensions (mm× mm × mm)	$A_{eff}\;(m^{2})$
**[13]**	1.8~2.5	32%	2.5~4	70 × 70 × 13.2	2.87 × 10^−3^
**[16]**	1.8~2.2	18.10%	7.8~10.2	190 × 100 × 1.6	1.55 × 10^−2^
**[17]**	2.3~2.63	13.40%	8.04~8.73	190.5 × 26 × 1.5	7.73 × 10^−3^
**[40]**	2~3.1	66%	2~3.6	35 × 50 × 1.6	1.70 × 10^−3^
**This work**	1.79~2.56	35.40%	6.2~9.34	138.6 × 47.7 × 1.57	9.34 × 10^−3^

## Data Availability

Data are contained within the article.
